# YopJ-Induced Caspase-1 Activation in *Yersinia*-Infected Macrophages: Independent of Apoptosis, Linked to Necrosis, Dispensable for Innate Host Defense

**DOI:** 10.1371/journal.pone.0036019

**Published:** 2012-04-26

**Authors:** Ying Zheng, Sarit Lilo, Patricio Mena, James B. Bliska

**Affiliations:** Department of Molecular Genetics and Microbiology, and Center for Infectious Diseases, Stony Brook University, Stony Brook, New York, United States of America; University of Maryland, United States of America

## Abstract

Yersinia outer protein J (YopJ) is a type III secretion system (T3SS) effector of pathogenic *Yersinia* (*Yersinia pestis*, *Yersinia enterocolitica* and *Yersinia pseudotuberculosis*) that is secreted into host cells. YopJ inhibits survival response pathways in macrophages, causing cell death. Allelic variation of YopJ is responsible for differential cytotoxicity in *Yersinia* strains. YopJ isoforms in *Y. enterocolitica* O:8 (YopP) and *Y. pestis* KIM (YopJ^KIM^) strains have high cytotoxic activity. In addition, YopJ^KIM^-induced macrophage death is associated with caspase-1 activation and interleukin-1β (IL-1β secretion. Here, the mechanism of YopJ^KIM^-induced cell death, caspase-1 activation, and IL-1β secretion in primary murine macrophages was examined. Caspase-3/7 activity was low and the caspase-3 substrate poly (ADP-ribose) polymerase (PARP) was not cleaved in *Y. pestis* KIM5-infected macrophages. In addition, cytotoxicity and IL-1β secretion were not reduced in the presence of a caspase-8 inhibitor, or in B-cell lymphoma 2 (Bcl-2)-associated X protein (Bax)/Bcl-2 homologous antagonist/killer (Bak) knockout macrophages, showing that YopJ^KIM^-mediated cell death and caspase-1 activation occur independent of mitochondrial-directed apoptosis. KIM5-infected macrophages released high mobility group protein B1 (HMGB1), a marker of necrosis, and microscopic analysis revealed that necrotic cells contained active caspase-1, indicating that caspase-1 activation is associated with necrosis. Inhibitor studies showed that receptor interacting protein 1 (RIP1) kinase and reactive oxygen species (ROS) were not required for cytotoxicity or IL-β release in KIM5-infected macrophages. IL-1β secretion was reduced in the presence of cathepsin B inhibitors, suggesting that activation of caspase-1 requires cathepsin B activity. Ectopically-expressed YopP caused higher cytotoxicity and secretion of IL-1β in *Y. pseudotuberculosis*-infected macrophages than YopJ^KIM^. Wild-type and congenic caspase 1 knockout C57BL/6 mice were equally susceptible to lethal infection with *Y. pseudotuberculosis* ectopically expressing YopP. These data suggest that YopJ-induced caspase-1 activation in *Yersinia*-infected macrophages is a downstream consequence of necrotic cell death and is dispensable for innate host resistance to a strain with enhanced cytotoxicity.

## Introduction

Induction of host cell death is a general and a very important outcome of pathogen infection, since cell death may facilitate pathogen clearance by removal of infected tissues, destruction of a pathogenic niche or up modulation of host immune responses [1], [2]. On the other hand, some pathogens subvert host immune responses by killing immune cells [3], [4]. Not only the death of the infected cells impacts the consequence of the battle between host immune system and pathogens, but also the choice of cell death pathway is important [5]. Apoptosis is characterized as having serial apoptotic caspase activation and as non-inflammatory. Apoptosis of immune cells such as macrophages is induced in response to infection by many pathogens, such as *Mycobacterium tuberculosis* and *Y. pseudotuberculosis* [6], [7], [8]. Unlike apoptosis, necrosis (also called oncosis, the term necrosis will be used here) is a form of traumatic death that results from acute cellular injury and is independent of caspase activation. Necrotic cell death is characterized by cell swelling, membrane rupture and release of inflammatory contents [9], [10].

A more recently characterized form of pro-inflammatory cell death that requires caspase-1 is termed pyroptosis [9]. The inflammasome is an intracellular sensor composed of NOD-like receptors (NLRs) that recognizes a variety of pathogen associated molecular patterns (PAMPs) and damage associated molecular patterns (DAMPs) and activates caspase-1, allowing for cleavage and secretion of cytokines such as IL-1β and IL-18 [11], [12]. Inflammasomes show specificity in signal sensing: the NLR NLRP1 (NALP1) inflammasome responds to *Bacillus anthracis* lethal toxin (LT) [13]; NLR NLRC4 (IPAF) recognizes flagellin from *Salmonella enterica* Typhimurium and *Legionella pneumophila*, which is delivered into host cells by specialized secretion systems in these pathogens [14], [15]; and NLR NLRP3 senses a group of structurally unrelated PAMPs and DAMPs, such as extracellular ATP, lipoproteins, double stranded RNA, potassium (K^+^) efflux, uric acid crystals, and pore-forming toxins from Gram positive bacteria *Listeria monocytogenes* and *Staphylococcus aureus* [16], [17], [18]. Pro-caspase-1 recruited by inflammasomes undergoes self-cleavage to give rise to an active form, which processes pro-IL-1β and pro-IL-18 to mature cytokines. Caspase-1 recruited to inflammasomes also induces cell death, but in this case cleavage of caspase-1 may not be required [19]. Thus, functionally distinct inflammasomes may form in cells in response to pathogen infection [19], [20]. Pyroptosis is defined as a caspase-1 dependent cell death, which morphologically exhibits DNA fragmentation, damaged cell membrane, and IL-18 and IL-1β release [9]. Pyroptosis occurs in macrophages infected with *Salmonella*, *Shigella* or *Fransicella* species, and can be blocked by caspase-1 inhibitor or by the use of caspase-1 deficient cells [21], [22], [23]. A forth type of cell death termed pyronecrosis has been observed in macrophages infected with *Shigella flexneri*, or *Neisseria gonorrhoeae*, or upon nigericin treatment. Pyronecrosis requires the NLR NLRP3 and the apoptosis-associated speck-like protein containing a CARD (ASC) adaptor, but not caspase-1 [24], [25], [26], [27], [28], [29]. Cell death during pyronecrosis can be blocked with cathepsin B inhibitors, suggesting a role for lysosome rupture [30].

Pathogenic *Yersinia* species (*Y. enterocolitica*, *Y. pestis* and *Y. pseudotuberculosis*) encode an injectisome-like T3SS that functions to translocate Yops into target cells [31]. Yop effector proteins disrupt cytoskeletal and signal transduction functions in infected immune cells to paralyze the host's anti-bacterial responses [31]. In turn, infected host cells can sense the *Yersinia* T3SS as a virulence-associated danger signal, leading to activation of caspase-1 [32], [33], [34], [35], [36], [37]. There are at least two distinct mechanisms of caspase-1 activation in response to the *Yersinia* T3SS. One mechanism requires channel or pore formation in the host cell plasma membrane by the T3SS, and is counteracted by several Yop effectors, including YopK [33], [34], [36], [37]. A second mechanism of caspase-1 activation that occurs in *Yersinia*-infected macrophages requires the effector YopJ (see below).

YopJ (YopP in *Y. enterocolitica*) is an acetyltransferase [38], [39] activated by the host-specific factor inositol hexakisphosphate [40]. YopJ binds to mitogen-activated protein (MAP) kinase kinases (MKKs) and inhibitor of nuclear factor kappa-B kinase beta (IKKβ) and transfers acetyl groups onto serine or threonine residues in the active sites of these kinases [38], [39]. Acetylation of MKKs and IKKβ by YopJ prevents their activation by upstream kinases, and effectively blocks signal transduction required for activation of MAP kinases and nuclear factor kappa B (NF-κB) transcription factors [38], [39]. As a result, YopJ activity inhibits transcription of pro-inflammatory cytokine and cell survival genes [41], [42]. Inhibition of survival gene expression by YopJ, combined with activation of apoptotic signaling from Toll-like receptor 4 (TLR4), results in cell death in macrophages infected with *Yersinia* [43], [44].

YopP-induced apoptosis in *Y. enterocolitica*-infected macrophages has been studied in detail and data suggest that the death signal is initiated from caspase-8 activation and further amplified through mitochondria and downstream caspases [41], [45]. Evidence supporting this model comes from studies showing that YopP-induced macrophage cell death is reduced by a pan-caspase inhibitor or a caspase-8 inhibitor, that cytochrome c is released from mitochondria, and that active caspase-3, -7 and -9 are detected [41], [45].

Different *Yersinia* strains exhibit a range of cytotoxic activities on macrophages and this heterogeneity has been linked to allelic variation of genes encoding YopJ/YopP proteins (Table 1) [32], [35], [46], [47], [48]. The presence of an Arg instead of a Ser at position 143 of YopP of *Y. enterocolitica* O:8 strains is associated with increased inhibition of IKKβ, enhanced suppression of NF-κB activation, and higher cytotoxicity in infected macrophages [46]. Translocation of YopP into host cells and binding to IKKβ was not affected by the polymorphism at position 143 [46]. YopJ proteins of *Y. pestis* and *Y. pseudotuberculosis* have Arg at residue 143 but in general have lower cytotoxicity than YopP of *Y. enterocolitica* O:8 due to comparatively reduced secretion and translocation into macrophages [47], [48]. Reduced secretion and translocation of YopJ proteins is caused by polymorphisms at positions 10 and 11, which are Ile-Ser in YopJ of *Y. pestis* and *Y. pseudotuberculosis* and Ser-Pro in YopP of *Y. enterocolitica* O:8 [47]. Ectopic expression of YopP of *Y. enterocolitica* O:8 in *Y. pseudotuberculosis* or *Y. pestis* results in attenuation of these strains in mouse models of infection [47], [49], which suggests that enhanced cytotoxicity may activate an innate host immune response to the pathogen.

**Table 1:** Amino acid polymorphisms that are associated with differences in translocation or IKKβ binding or inhibition activities between different YopJ/YopP isoforms.

	Amino acid position				
	Translocation		IKKβ binding or inhibition		
Isoform	10	11	143	177	206
YopP^08^	Ser	Pro	Arg	Leu	Glu
YopJ^KIM^	Ile	Ser	Arg	Leu	Glu
YopJ^YPTB^	Ile	Ser	Arg	Phe	Glu
YopJ^CO92^	Ile	Ser	Arg	Phe	Lys

Additional polymorphisms among YopJ proteins in *Y. pestis* and *Y. pseudotuberculosis* have been identified that are responsible for differences in macrophage cytotoxicity [32]. An isoform of YopJ found in *Y. pestis* molecular group 2.MED strains such as KIM (YopJ^KIM^) have high cytotoxic activity and contain a Leu at position 177 and a Glu at position 206 [32]. Low activity YopJ isoforms found in other *Y. pestis* strains (e.g. molecular group ORI.1 isolate CO92) have Phe at residue 177 and Lys at position 206 [32]. The YopJ isoform in *Y. pseudotuberculosis* has a single change relative to YopJ^KIM^, Phe at residue 177, and has intermediate cytotoxic activity in macrophages [32]. The increased cytotoxic activity of YopJ^KIM^ as compared to YopJ^CO92^ could be correlated with enhanced binding to IKKβ, and enhanced inhibition of NF-κB activation [32].

Detailed studies of the features of death in host cells infected with *Yersinia* strains that encode YopJ isoforms with high cytotoxic activity have yielded evidence that pro-inflammatory modes of destruction may be activated in addition to apoptosis. For example, murine dendritic cells infected with *Y. enterocolitica* O:8 undergo YopP-dependent necrotic cell death [50]. In addition, infection of murine macrophages with *Y. pestis* KIM results in YopJ-dependent activation of caspase-1 and secretion of high levels of IL-1β [32], [35]. Human monocytes infected with KIM also secrete high levels of IL-1β [51]. Caspase-1 is not required for YopJ^KIM^-induced cell death but is important for secretion of IL-1β from macrophages [35]. K^+^ efflux, NLRP3 and ASC were shown to be important for IL-1β secretion in macrophages infected with *Y. pestis* KIM [32]. However, the morphological features and the mechanism of YopJ^KIM^-induced macrophage death have not determined, and the mechanistic link between cytotoxicity and caspase-1 activation has not been established.

In this study, we examined the mechanism of cell death and caspase-1 activation in macrophages infected with *Y. pestis* KIM. The results suggest that YopJ^KIM^ induces necrotic cell death in macrophages, which triggers a cathepsin B-dependent pathway of caspase-1 activation. In addition, we show that macrophages infected with *Y. pseudotuberculosis* ectopically expressing YopP are efficiently killed and secrete high levels of IL-1β. However, infection of caspase-1-deficient mice revealed that increased host resistance to a *Y. pseudotuberculosis* YopP-expressing strain endowed with enhanced cytotoxicity does not require caspase-1.

## Results

### Caspase-3/7 activity is low in KIM5-infected macrophages

**Figure 1:**
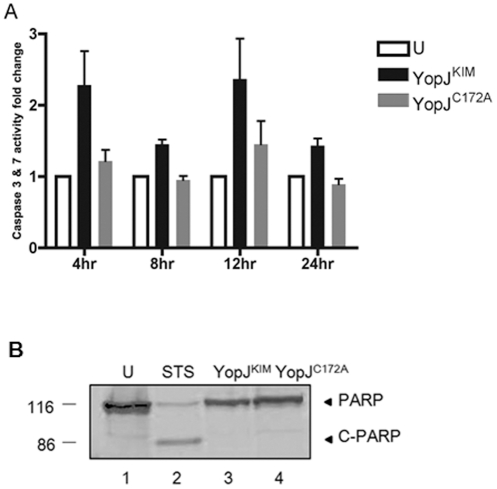
Caspase-3/7 activity is low in KIM5-infected macrophages. (A) BMDMs were left uninfected (U) or infected with *Y. pestis* strains expressing YopJ^KIM^ or YopJ^C172A^ in 96-white walled tissue culture plates. Caspase-3/7 activity was measured 4, 8, 12 or 24 hr post-infection with fluorometer. The results from three independent experiments were averaged and are shown as fold change compared to uninfected cells. Error bars represent standard deviations. Differences in caspase-3/7 activities between uninfected and infected cells were not significant as determined by two way ANOVA (B) BMDMs were left uninfected (U) or infected with *Y. pestis* strains expressing YopJ^KIM^ or YopJ^C172A^ or treated with 1 µM of staurosporine (STS) for 16 hr. Macrophage lysates were collected and analyzed by PARP immunoblotting. Sizes of molecular weight standards (kDa) are shown on the left. Positions of full length PARP and cleaved PARP (c-PARP) are showed on right.

Activation of apoptotic caspases, such as caspase-3, -7 and -9, has been detected in macrophages undergoing YopP-dependent cell death in response to *Y. enterocolitica* infection [45]. Caspase-3/7 activity assay was performed to examine apoptotic caspase activation at 4, 8, 12 and 24 hours post-infection in macrophages undergoing YopJ^KIM^-induced cell death following infection with *Y. pestis*. Murine bone marrow derived macrophages (BMDMs) were infected at a multiplicity of infection (MOI) of 10 for 20 min. The tissue culture media was then supplemented with gentamicin for the remaining period of incubation to prevent growth of extracellular bacteria [32], [35]. This “low MOI" infection procedure has previously been shown to cause cytotoxicity, activation of caspase-1, and high level secretion of IL-1β in macrophages [32], [35]. As controls, some macrophages were left uninfected or infected with a *Y. pestis* strain expressing catalytically inactive YopJ^C172A^. Caspase-3/7 activity in KIM5-infected macrophages was higher as compared to the controls at all time points, but the difference was not significant (Figure 1A). Lysates of macrophages infected as in Figure 1 were subjected to immunoblotting to detect cleavage of the caspase-3 substrate PARP. Lysates of control macrophages treated with staurosporin, a strong inducer of apoptosis, were analyzed in parallel. The 86 kDa cleaved PARP (c-PARP) fragment was not detected lysates of KIM5-infected cells, but it was seen in staurosporin-treated cell lysates (Figure 1B). These results show that apoptotic caspases are not strongly activated in macrophages undergoing YopJ^KIM^-induced cell death.

### Apoptotic signaling through caspase-8 and mitochondria is dispensable for KIM5-induced macrophage death

**Figure 2:**
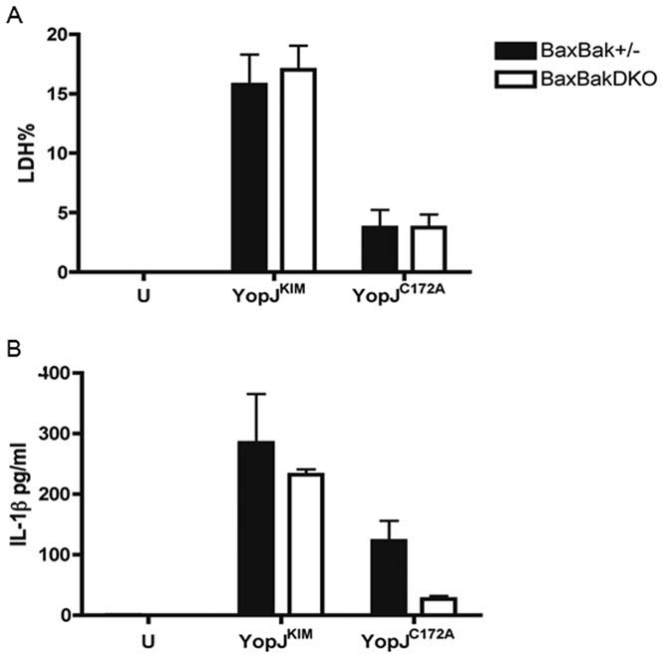
Mitochondrial-induced apoptosis is not required for KIM5-induced macrophage death and IL-1β secretion.

It has been shown that YopP-induced apoptosis is initiated from caspase-8 in macrophages and dendritic cells infected with *Y. enterocolitica*, and can be blocked by caspase-8 or pan-caspase inhibitors [45], [52]. A detailed study detected BH3 domain only protein (Bid) truncation before cytochrome c release and apoptosome activation, which suggests that the death signal coming from caspase-8 may require mitochondria and is amplified through caspase -3,-7 and -9 cleavage [45]. Bcl-2 family members Bax and Bak play a central role in controlling mitochondrial-dependent apoptosis [53]. Bax and Bak when activated create a channel in the mitochondrial membrane, releasing cytochrome C to activate the apoptosome. In order to test if the YopJ^KIM^-dependent death signal goes through mitochondria, we infected Bax^−/−^Bak^−/−^ macrophages with KIM5, using heterozygous Bax^+/−^Bak^+/−^ cells as the control. The Bax^−/−^Bak^−/−^ macrophages have been shown to be fully defective for mitochondrial-induced apoptosis [53]. Cell death was measured by lactate dehydrogenase (LDH) release assay and secreted IL-1β was measured by enzyme-linked immunosorbent assay (ELISA) at 24 hr post infection. No significant differences in YopJ^KIM^-induced cell death or IL-1β release could be identified between Bax^−/−^Bak^−/−^ or Bax^−/+^Bak^−/+^ macrophages (Figure 2A and B).

**Figure 3:**
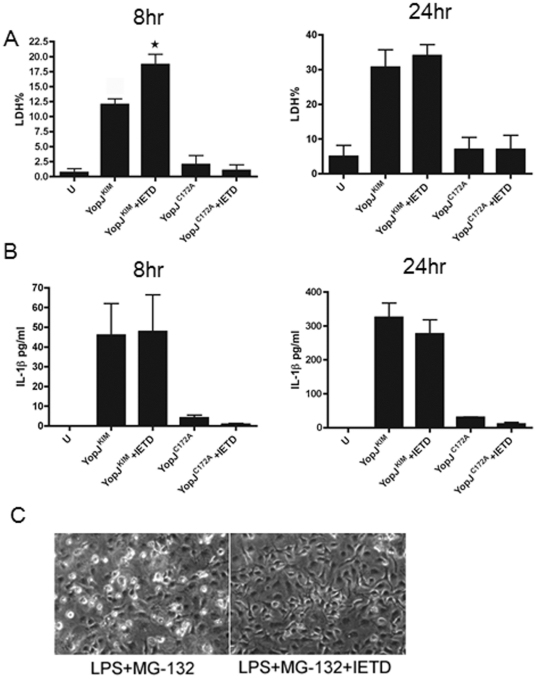
Caspase-8 activity is dispensable for KIM5-triggered macrophage death and IL-1β secretion. BMDMs were treated with 40 µM caspase-8 inhibitor Z-IETD (IETD) or vehicle 1 hr prior to infection. The BMDMs were then infected with *Y. pestis* strains expressing YopJ^KIM^ or YopJ^C172A^ or left uninfected (U). Infected cells were maintained in the presence of Z-IETD or the vehicle for the remainder of the experiment. At 8 hr or 24 hr post-infection, supernatants were collected and LDH release (A) and IL-1β (B) were measured. Results shown are averages from three independent experiments. Error bars represent standard deviations. ★, P<0.05 as determined by one way ANOVA compared to the YopJ^KIM^ infection without inhibitor condition. (C) BMDMs were treated with 5 µM of MG-132 in the presence or absence of 40 µM Z-IETD for 30 min, followed by 1 µg/ml of LPS for 3 hrs. Representative phase images of the treated BMDMs were captured by digital photomicroscopy.

To determine if caspase-8 is required for cell death in KIM5-infected macrophages, cells were exposed to the caspase-8 inhibitor IETD. IETD treatment did not significantly reduce macrophage death or IL-1β secretion after 8 or 24 hours of infection with KIM5 (Figure 3A and B). IETD treatment did increase cytotoxicity in KIM5-infected macrophages at the 8 hours time point, but this effect was not seen at the 24 hour time point (Figure 3A and B) or in macrophages infected with *Y. pestis* expressing YopJ^C172A^ (Figure 3A and B). As a control, IETD treatment was shown to effectively block cell death in macrophages caused by treatment with lipopolysaccharide (LPS) and the proteasome inhibitor MG-132 (Figure 3C). Thus, apoptotic signaling through caspase-8 and mitochondria is not required for YopJ^KIM^-induced macrophage death.

### KIM5-infected macrophages exhibit necrotic features

**Figure 4:**
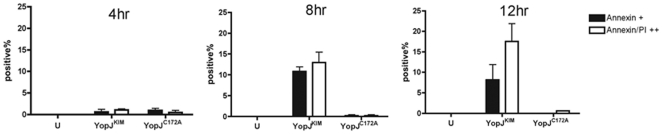
KIM5-infected macrophages have necrotic morphology as shown by Annexin V staining and PI uptake assay. BMDMs were seeded on glass coverslips in a 24-well plate and infected with *Y. pestis* strains expressing YopJ^KIM^ or YopJ^C172A^ or left uninfected (U). Annexin V staining and PI uptake assay was performed at 4 hr, 8 hr or 12 hr post-infection. Representative images were captured by digital photomicroscopy. Average percentages of Annexin V positive or Annexin V/PI double positive cells as counted from three random fields in three independent experiments are shown. Error bars represent standard deviations. Difference in values of single or double positive cells were not significant as determined by one way ANOVA.

To characterize the plasma membrane integrity of KIM5-infected macrophages, we performed Annexin V staining/propidium iodide (PI) uptake assay at different times (4, 8 and 12 hr post infection) and analyzed the results by fluorescence microscopy. Macrophages infected with *Y. pestis* expressing YopJ^C172A^ were analyzed in parallel as a control. As summarized in Figure 4, two populations of cells that were either Annexin V single positive (apoptotic) or Annexin V/PI double positive (necrotic) were detected beginning at 8 hr post infection with KIM5. The number of Annexin V single positive cells (∼11% at 8 hr) declined slightly to 8% of total by 12 hr post infection. The double positive population (∼13% at 8 hr) increased with time to reach 17% of total by 12 hr (Figure 4).

**Figure 5:**
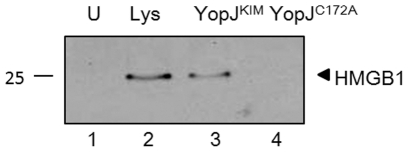
HMGB1 is released from KIM5-infected macrophages. BMDMs were infected with *Y. pestis* strains expressing YopJ^KIM^ or YopJ^C172A^ or left uninfected (U). Medium from infected macrophages was collected at 24 hr post infection and immunoblotted for HMGB1. Total cell lysate (Lys) was used as a positive control. Position of molecular weight standard (kDa) is shown on the left.

Release of HMGB1, a chromatin protein, can be used to differentiate apoptosis from necrosis [54]. Immunoblotting was used to detect HMGB1 in culture supernatants of macrophages following 24 hr infection with KIM5 or KIM5 expressing YopJ^C172A^. As shown in Figure 5, HMGB1 was released from macrophages infected with *Y. pestis* expressing YopJ^KIM^ but not YopJ^C172A^ (lanes 3 and 4, respectively). Together, these results suggest that a population of KIM5-infected macrophages undergo necrosis.

### KIM5-infected necrotic macrophages contain active caspase-1

**Figure 6:**
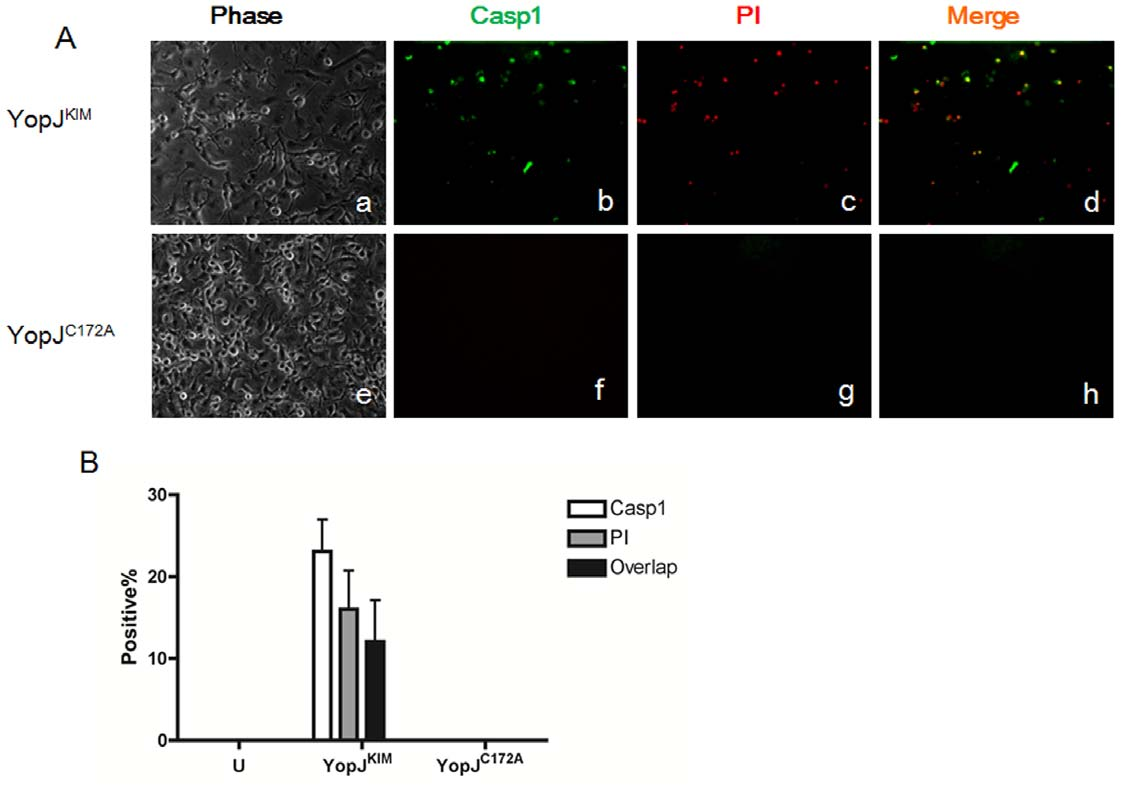
KIM5-infected necrotic macrophages contain active caspase-1. BMDMs were seeded on glass coverslips in a 24-well plate and left uninfected (U) or infected with *Y. pestis* strains expressing YopJ^KIM^ or YopJ^C172A^. FAM-YVAD-FMK was added at 9 hr post infection to stain for active caspase-1 and PI uptake assay was performed immediately before microscopic analysis. (A) Representative images of phase, active caspase-1 (green) and PI uptake (red) signals captured by digital photomicroscopy are shown in a-c and e-g, respectively. Panels d and h show merged images of green and red signals. (B) Average percentages of BMDMs positive for active caspase-1, PI or both signals was calculated (∼100–300 cells per field) from three random fields in three independent experiments. Error bars represent standard deviations.

As both cell death and IL-1β release require YopJ^KIM^ and they display the same trends, caspase-1 activation and cell death appear to be related. It is possible that necrotic cell death triggers caspase-1 activation [35]. To determine if necrotic cell death and caspase-1 activation could be correlated at the single cell level, infected macrophages were analyzed by microscopy after labeling for active caspase-1 and PI uptake. Representative images of macrophages infected with KIM5 or *Y. pestis* expressing YopJ^C172A^ for 9 hr are shown in Figure 6A, and a summary of the percentages of cells that were positive for one or both signals is shown in Figure 6B. The percentages of KIM5-infected macrophages that were caspase-1 positive, PI positive and double positive were not significantly different (Figure 6B), which suggests that membrane damage in necrotic cells is associated with caspase-1 activation.

### RIP1 is not required for YopJ^KIM^-induced cell death or IL-1β secretion

**Figure 7:**
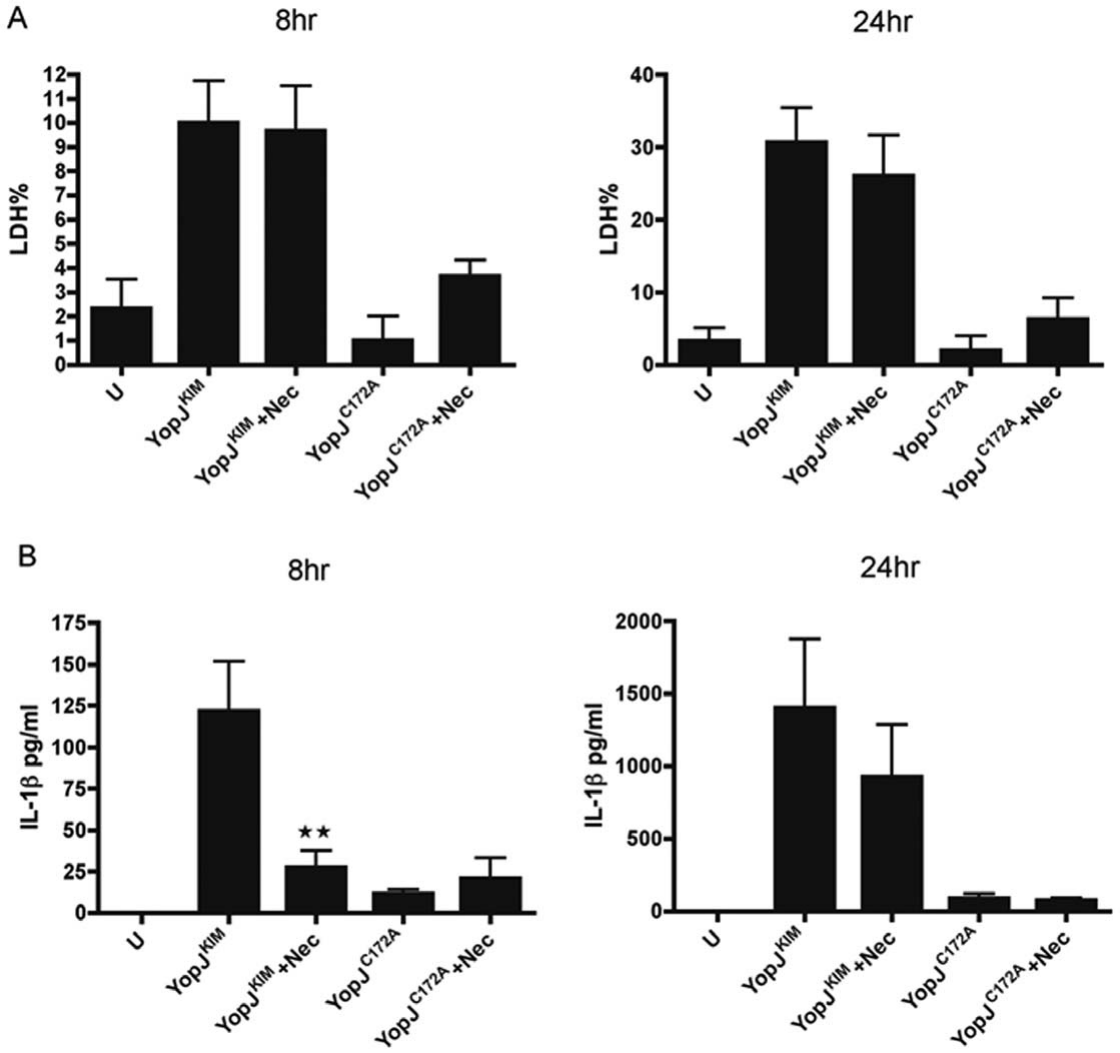
RIP1 is not required for YopJ^KIM^-induced cell death or IL-1β secretion. BMDMs were treated with 30 µM RIP1 inhibitor necrostatin-1 (Nec) or vehicle 1 hr prior to and during infection. BMDMs were infected with *Y. pestis* strains expressing YopJ^KIM^ or YopJ^C172A^ or left uninfected (U). Supernatants were collected and LDH release (A) and secreted IL-1β (B) were measured at 8 hr or 24 hr post-infection from three independent experiments. Results shown are averages and error bars represent standard deviations (★★, *P*<0.01 as determined by one way ANOVA as compared to YopJ^KIM^ no inhibitor).

YopP-mediated dendritic cell death in response to *Y. enterocolitica* infection is reduced by treatment with geldanamycin, a heat shock protein 90 (Hsp90) inhibitor, which promotes RIP1 degradation [52]. Furthermore, YopP-induced dendritic cell death is partially independent of caspases and exhibits necrotic features [50], which are similar to our findings with YopJ^KIM^. To test if RIP1 is involved in KIM5-induced macrophage death, we treated cells with the specific RIP1 inhibitor necrostatin-1, which blocks necrosis in many cell types [55]. The treated macrophages were then infected with KIM5 or *Y. pestis* expressing YopJ^C172A^ for 8 or 24 hours. Necrostatin-1 did not significantly reduce cell death at either time point in KIM5-infected macrophages (Figure 7A). IL-1β release was significantly reduced at 8 hr, but not at 24 hr post infection with KIM5 (Figure 7B). From these results we conclude that RIP1 is not required for YopJ^KIM^-induced cell death or IL-1β secretion, however it may enhance IL-1β secretion at early infection times.

### ROS are not required for cytotoxicity or IL-1β secretion in macrophages infected with KIM5

**Figure 8:**
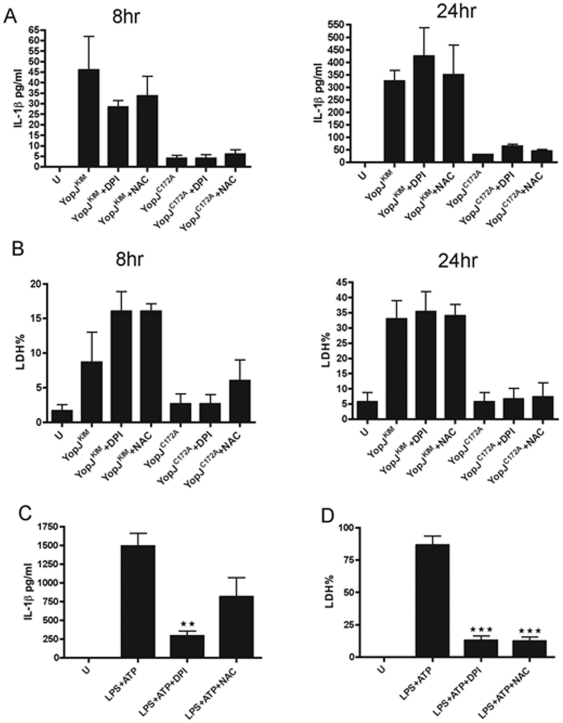
ROS are not required for cytotoxicity or IL-1β secretion in macrophages infected with KIM5. BMDMs were treated with 10 µM of DPI or 10 mM of NAC for 2 hours or left untreated. (A and B) BMDMs were infected with *Y. pestis* strains expressing YopJ^KIM^ or YopJ^C172A^ or left uninfected (U). Supernatants were collected at 8 hr and 24 hr post-infection and analyzed by IL-1β ELISA (A) or LDH release assay (B). (C and D) BMDMs treated or not with DPI or NAC as above were exposed to 50 ng/ml of LPS for 3 hr. The treated BMDMs were then exposed to 5 mM ATP for 1 hr to activate pyroptosis. Supernatants were tested by IL-1β ELISA (C) or LDH release assay (D). Results shown are the averages from three independent experiments. Error bars represent standard deviations (★★, *P*<0.01; ★★★, *P*<0.001, determined by one way ANOVA as compared to LPS+ATP no inhibitor).

NLRP3 is important for IL-1β secretion in KIM5-infected macrophages [32]. NLRP3 senses several structurally unrelated PAMPs and DAMPs that share the common property of inducing ROS [56]. It has therefore been proposed that ROS is a major signal detected by NLRP3 [56]. The nicotinamide adenine dinucleotide phosphate-oxidase (NADPH) inhibitor diphenyleneiodonium sulfate (DPI) and the radical scavenger N-acetylcysteine (NAC) were used to examine the importance of ROS for cytotoxicity and IL-1β secretion in KIM5-infected macrophages. Pretreatment of macrophages with DPI or NAC did not reduce IL-1β release or cell death following KIM5 infection of macrophages for 8 or 24 hr (Figure 8A and 8B). As a control, LPS-stimulated macrophages were exposed to DPI or NAC and NLRP3-dependent pyroptosis was induced by ATP treatment. The amount of IL-1β released was significantly reduced by DPI and cytotoxicity was significantly reduced by DPI and NAC (Figure 8C and 8D). Therefore, ROS are not required for YopJ^KIM^-induced cell death or IL-1β secretion.

### Inhibitors of cathepsin B reduce caspase-1 activation in macrophages infected with KIM5

**Figure 9:**
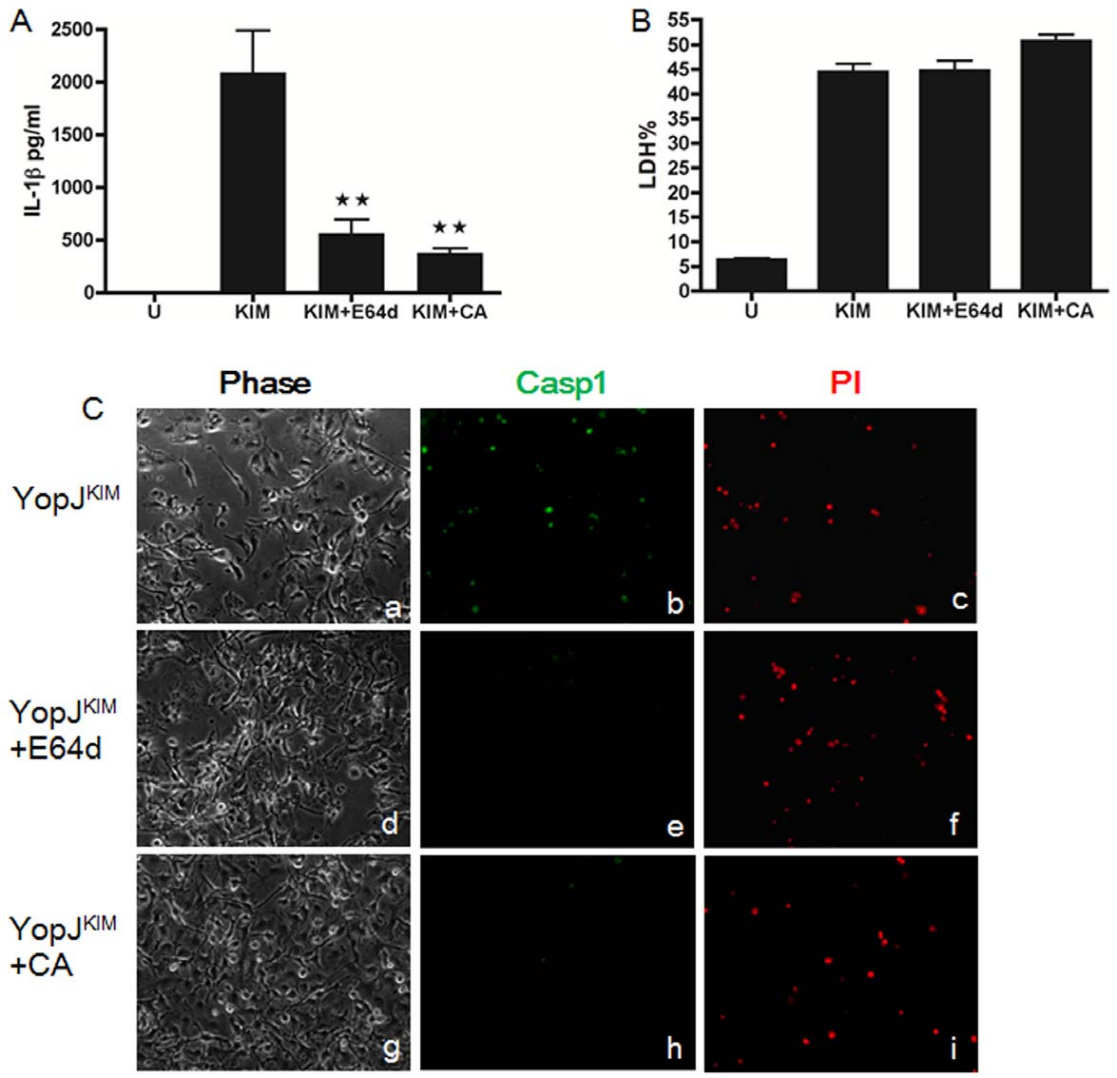
Inhibitors of cathepsin B reduce caspase-1 activation in macrophages infected with KIM5. BMDMs were left untreated or treated with 25 µM of E64d or CA-074 Me (CA) for 1 hr. Following infection with *Y. pestis* strains expressing YopJ^KIM^ in the absence or presence of the inhibitors, supernatants were collected (A, B) or microscopic assay was performed (C). IL-1β ELISA (A) and LDH release assay (B) was done on supernatants collected 24 hr post-infection. Results shown are the averages from three independent experiments. Error bars represent standard deviations. (★★, *P*<0.01 as determined by one way ANOVA as compared to infection in absence of inhibitor) (C) Infected BMDMs on coverslips were incubated with FAM-YVAD-FAM 9 hr post-infection stained for active caspase-1 (green) for 1 hr and PI uptake (red) immediately before observation. Representative images of phase, green and red signals were captured by digital photomicroscopy.

Lysosomal rupture leads to release of lysosome-localized protease cathepsin B into the cytoplasm, which directly or indirectly activates NLRP3/caspase-1 [57]. We examined the lysosome rupture pathway using the cathepsin inhibitor E64d and specific cathepsin B inhibitor CA-074-Me. Treatment of macrophages with either of these two inhibitors during a 24 hr KIM5 infection resulted in a significant decrease in secretion of IL-1β (Figure 9A) but had no effect on cytotoxicity (Figure 9B). Microscopic imaging of KIM5-infected macrophages after straining them for active caspase-1 and PI uptake showed that E64d and CA-074-Me blocked caspase-1 activation (Figure 9C). These results suggest that cathepsin B activity is required for YopJ^KIM^-mediated activation of caspase-1.

### Enhanced YopP-mediated macrophage cell death is associated with elevated levels of IL-1β release

**Figure 10:**
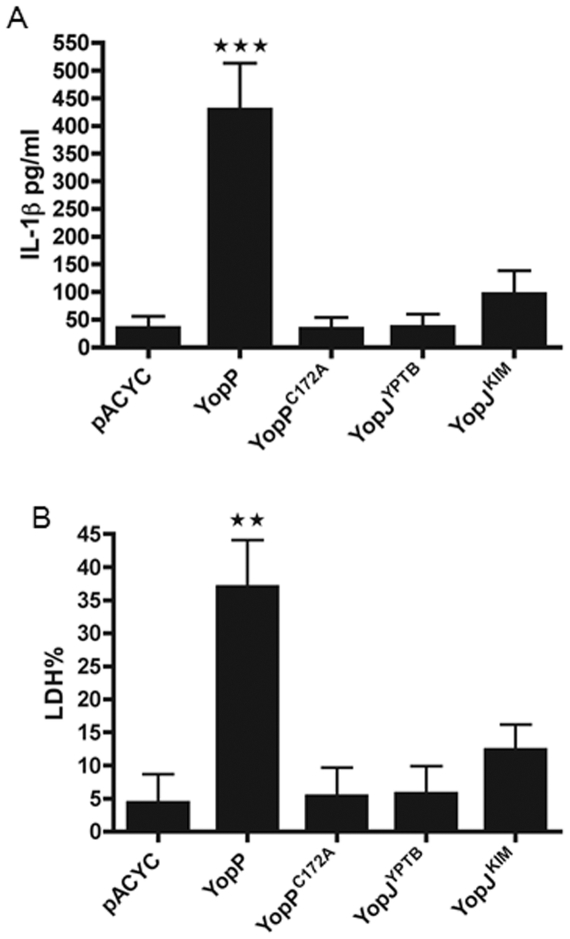
Enhanced YopP-mediated macrophage cell death is associated with elevated levels of IL-1β release. *Y. pseudotuberculosis* IP26 (*ΔyopJ*) carrying the empty pACYC184 plasmid (pACYC) or pACYC184 encoding the indicated YopP or YopJ isoforms was used to infect BMDMs. Twenty four hours post-infection, medium was collected for IL-1β ELISA (A) and LDH release assay (B). Results shown are the averages from three independent experiments. Error bars represent standard deviations (★★, *P*<0.01; ★★★, *P*<0.001 as determined by one way ANOVA as compared to pACYC condition).

Higher levels of cell death are observed when dendritic cells are infected with *Y. pseudotuberculosis* ectopically expressing YopP as compared to the native isoform YopJ^YPTB^ [47]. Two amino acid polymorphisms in the N-terminal region of YopP specify increased secretion, translocation and cytotoxic activity as compared to YopJ^YPTB^ (Table 1) [47]. Macrophages were infected with *Y. pseudotuberculosis* ectopically expressing YopP to determine if the enhanced cell death caused by this isoform is correlated with higher caspase-1 activation and IL-1β secretion. Macrophages were also infected with *Y. pseudotuberculosis* expressing catalytically inactive YopP (YopP^C172A^), the native isoform YopJ^YPTB^, or YopJ^KIM^. The different isoforms were expressed from a low copy plasmid (pACYC184) in a *Y. pseudotuberculosis ΔyopJ* mutant (IP26). Higher cytotoxicity and IL-1β release was detected in macrophages infected with *Y. pseudotuberculosis* expressing YopP as compared to the other isoforms or the control strain with the empty vector (Figure 10A and B). In ranking the different isoforms YopP had the highest cytotoxicity, YopJ^YPTB^ the lowest killing effect, and YopJ^KIM^ was intermediate. IL-1β release followed the same order YopP>YopJ^KIM^>YopJ^YPTB^.

### Caspase-1 is not required for innate host protection against *Yersinia* endowed with enhanced cytotoxicity

**Figure 11:**
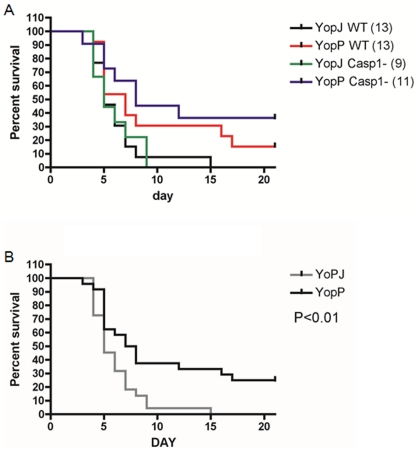
Caspase-1 is not required for innate host protection against *Yersinia* endowed with enhanced cytotoxicity. (A) Six to eight-week old Casp1*^+/+^* (wild type, WT) or Casp1*^−/−^* (Casp1-) C57BL/6J mice were infected orogastrically with 1×10^9^ CFU of *Y. pseudotuberculosis* IP26 carrying pACYC184 encoding YopP or YopJ^YPTB^. Mouse survival was monitored for 21 days. Results shown are pooled from two independent experiments. Total numbers of mice infected are shown in parenthesis. (B) Data from (A) are reformatted by grouping mice according to infecting strain. Significant difference between survival curves was determined by log rank test.

*Y. pseudotuberculosis* or *Y. pestis* strains ectopically expressing YopP are attenuated in orogastric [47] or bubonic [49] models of mouse infection, respectively. The basis for attenuation of *Yersinia* strains endowed with enhanced cytotoxicity is not known, but it appears to result from an increased innate immune response and does not require CD8 T cell activation [47], [49]. To determine if activation of caspase-1 is important for the increased innate immune response to *Yersinia* endowed with enhanced cytotoxicity, Casp1^+/+^ or Casp1^−/−^ C57BL/6 mice were orogastrically infected with *Y. pseudotuberculosis* ectopically expressing YopP. Control mice were infected with *Y. pseudotuberculosis* ectopically expressing YopJ^YPTB^. Mouse survival was recorded over 21 days. As shown previously [47] more Casp1*^+/+^* mice infected with the YopP-expressing strain survived as compared to mice infected with YopJ^YPTB^-expressing bacteria (Figure 11A). However, Casp1*^−/−^* mice also showed enhanced survival following challenge with YopP-expressing *Y. pseudotuberculosis*, indicating that caspase-1 is dispensable for the increased innate immune response to *Yersinia* with enhanced cytotoxicity. When the results were grouped according to the infecting strain while ignoring mouse genotype (Figure 11B), the survival of mice infected with *Y. pseudotuberculosis* expressing YopP was significantly higher than the mice infected with bacteria expressing YopJ^YPTB^ (P<0.01).

## Discussion

Many pathogens activate inflammasomes/caspase-1 in macrophages, underscoring the general importance of this pathway for host sensing of PAMPs and DAMPs and activating innate immune responses [58]. We, and others, have been investigating different mechanisms of inflammasome/caspase-1 activation in macrophages infected with pathogenic *Yersinia* species [32], [33], [34], [35], [36], [37]. *Yersinia*-mediated caspase-1 activation in macrophages can occur by several different mechanisms. Insertion of T3SS translocation channels or pores in the macrophage cell plasma membrane appear to activate caspase-1 and cause pyroptosis [33], [34], [36]. Priming of macrophages with LPS followed by *Yersinia* infection can redirect apoptosis to pyroptosis [37]. In both of the aforementioned cases YopJ is not required for activation of caspase-1. A third mechanism of caspase-1 activation that occurs in naive macrophages infected with *Yersinia* requires YopJ catalytic activity [32], [35], [36].

YopJ inhibits NF-κB and MAPK pathways that activate transcription of cell survival genes, promoting macrophage cell death in response to apoptotic signaling from TLR4 [43], [59]. In addition, suppression of the NF-κB pathway by YopJ [32] or genetic or pharmacological inhibition of IKKβ [60] triggers TLR4-dependent activation of caspase-1. The conditions used for infection of macrophages with *Yersinia* affect the outcome of YopJ-mediated caspase-1 activation. Incubation of macrophages with *Y. pseudotuberculosis* under conditions of high MOI (20) and extended contact with extracellular bacteria (1 hr) results in rapid activation of caspase-1 but IL-1β release is undetectable and caspase-1 activation does not depend on NLRP3 nor ASC [36]. Infection of macrophages with *Y. pestis* KIM5 under low MOI (10) and short contact time (20 min) results in delayed caspase-1 activation, high level of IL-1β release and cytotoxicity in a YopJ dependent manner [32], [35]. In addition, NLRP3 and ASC are important for IL-1β release from macrophages infected with KIM5 under the low MOI procedure [32]. As an outcome of YopJ blocking the NF-κB pathway, less pro-IL-1β would be synthesized in macrophages, but processing of even a small pool of pro-IL-1β by active caspase-1 can lead to detectable released IL-1β. In the low MOI infection conditions used here, it is likely that low amounts of YopJ are injected into macrophages, resulting in a delay in cell death and caspase-1 activation in macrophages. This infection condition may allow macrophages sufficient time to synthesize pro-IL-1β before cell death occurs.

To investigate the mechanism of YopJ^KIM^-induced caspase-1 activation and its connection to cell death in macrophages, we first investigated the importance of apoptosis. The low caspase-3/7 activity (Figure 1) and the dispensable role for caspase-8 (Figure 3) in cell death are consistent with the idea that apoptosis is not strongly activated in KIM5-infected macrophages under our experimental conditions. A previous study in which KIM5 was used to infect macrophages reported relatively high levels of caspase-3/7 and caspase-8 activity [61]. Spinner et al [61] used murine J774A.1 cells as well as an MOI of 50, which could explain why higher caspase activity was detected as compared to our results. Additional evidence for caspase-8 activation in *Yersinia*-infected macrophages comes from Bid cleavage assays. Caspase-8 cleavage has been detected in *Yersinia* infected dendritic cells and in macrophages treated with LPS/MG-132 to activate apoptosis [45], [52], [59]. Caspase-8 has been reported to process pro-IL-1β after stimulation of TLR3/TLR4 signaling in macrophages [62], but in our studies a caspase-8 inhibitor did not decrease IL-1β release in KIM5-infected macrophages (Figure 3). Participation of mitochondrial-induced apoptosis in death of macrophages infected with *Y. enterocolitica* has been implicated by the observed release of cytochrome *c* [45]. However, YopP-induced cell death in dendritic cells was not inhibited by overexpression of Bcl-2 [50]. In our studies the use of Bax/Bak knockout BMDMs would mimic Bcl-2 over-expression by preventing pore formation on the mitochondrial membrane, however loss of Bax and Bak did not decrease cell death or IL-1β secretion in KIM5-infected infected macrophages (Figure 2).

Necrosis releases inflammatory cell contents, which is consistent with proinflammatory cytokine production in KIM5-infected macrophages [35]. Annexin V/PI staining was performed to observe macrophage plasma membrane integrity over time. Annexin V single positive cells representing the early apoptosis population occurred in parallel with Annexin V/PI double positive cells representing the late apoptosis or necrosis population (Figure 4). The presence of the two populations indicates that apoptosis and necrosis may coincide. However, cells with the double positive phenotype may not arise from necrosis, especially in cell culture when apoptotic cells are not engulfed by bystander phagocytes, but rather from “secondary necrosis" [10]. Thus, to be sure that KIM5-infected macrophages are dying of necrosis, accumulation of more evidence is needed.

HMGB1 release is a very distinctive marker of necrosis [54]. The observed release of HMGB1 from KIM5-infected macrophages (Figure 5) strongly suggests that these cells are dying of necrosis. Although HMGB1 can be passively secreted by activated dendritic cells and macrophages [63], infection of macrophages with *Y. pestis* expressing YopJ^C172A^, which would activate macrophages through LPS-TLR4 signaling, did not result in HMGB1 release (Figure 5). To determine if released HMGB1 could be important for cell death and activation of caspase-1, medium from KIM5-infected macrophages was transferred to naive uninfected macrophages. However, the conditioned medium did not increase IL-1β release or cell death in uninfected macrophages (Figure S1). Although HMGB1 has been shown to stimulate pro-inflammatory cytokine production [64], [65], [66], it is unlikely that HMGB1 interacts with TLR4 to promote IL-1β production and cell death in our system.

Since necrotic cells release inflammatory cytokines and KIM5-infected macrophages showed necrotic properties (Figure 4 and 5), we performed caspase-1/PI staining to see if caspase-1 activation takes place in necrotic cells (Figure 6). The highly overlapped caspase-1/PI positive cell population supports the idea that these two events occur in same cells. However, it is difficult to discriminate if necrosis occurs earlier than caspase-1 activation. The results of previous LDH and IL-1β time course release assays showed LDH release 4 hr ahead of IL-1β secretion, suggesting that cell death may happen earlier than caspase-1 activation [35].

As macrophages infected with KIM5 seem to die by necrosis, and cell death initiated earlier than IL-1β release [35], we hypothesized that necrosis could activate the inflammasome/caspase-1. We tried to blocked necrosis through use of the RIP1 inhibitor necrostatin-1. RIP1 has been identified as an important mediator of non-apoptotic death in many cell types. When caspase-8 activity is inhibited, preventing cleavage of RIP1, RIP1 positively activates a necrotic (necroptosis) pathway [67]. Cell death triggered with Fas ligand (FasL) or tumor necrosis factor-α (TNF-α) through caspase-8 activation, combined with pan-caspase or caspase-8 inhibitor treatment, is RIP1 dependent and could be prevented by specific RIP1 inhibitor necrostatin-1 [68], [69], [70]. In macrophages after TLR4 stimulation, when the cell NF-κB survival signaling pathway and caspase-8 activation are inhibited, RIP1 causes necrosis [71], [72], [73]. RIP1 has also been implicated in death of dendritic cells infected with *Y. enterocolitica* O:8 strain WA-314, and the same group obtained evidence that dendritic cells could die by necrosis in the same infection conditions [50], [52]. However, in our infection model, the RIP1 specific inhibitor necrostatin-1 did not reduce cell death (Figure 7). Treatment with necrostatin did inhibit IL-1β release at 8 hours post infection (Figure 7), which may due to lack of interaction between RIP1 and the NF-κB pathway [71]. RIP3 has also been implicated in necrosis, and although it is not clear if RIP3 and RIP1 can form a heterodimer, RIP3 alone could induce necrosis [74], [75], [76]. It would be interesting to test RIP3 knockout BMDMs in the future to determine the role of this kinase in YopJ^KIM^-induced cell death and caspase-1 activation.

Two recent studies discovered that necrosis could activate the inflammasome/caspase-1 [77], [78]. In the study of Iyer et al., mitochondrial ATP release from necrotic cells activated NLRP3/caspase-1 in LPS primed macrophages through P2X_7_ receptor [78]. This pathway does not occur in our model, since we have shown that P2X_7_ receptor is not required for secretion of IL-1β in KIM5-infected macrophages [32].

NLRP3 and ASC were important for secretion of IL-1β from KIM5-infected macrophages, although these inflammasome components were dispensable for cell death [32]. Consistent with an important role for NLRP3 and ASC in caspase-1 activation was the observation that exogenous K^+^ inhibited secretion of IL-1β from KIM5-infected macrophages. Specifically, extracellular K^+^, but not Na^+^, down regulated IL-1β release in KIM5-infected macrophages, suggesting that NLRP3 activation requires a low concentration of intracellular K^+^ [32]. Low intracellular K^+^ levels could result from intracellular K^+^ passing through ATP-sensitive K^+^ channels (such as P2X_7_), or by its release from dying cells [56], [79]. As mentioned above P2X_7_ receptor is not required for IL-1β release in KIM5-infected macrophages, suggesting that pore formation in necrotic macrophages may allow K^+^ efflux.

As NLRP3 can recognize ROS generation, or lysosome rupture leading to caspase-1 activation, we tested each of these processes for their importance in IL-1β secretion in KIM5-infected macrophages. With respect to the ROS generation model, most pathogens that activate caspase-1 through NLRP3 induce ROS generation and in many cases, K^+^ efflux occurs simultaneously [56]. However, two ROS inhibitors, DPI and NAC, had no significant effect on IL-1β release or cell death in KIM5-infected macrophages (Figure 8). These inhibitors did reduce pyroptosis of macrophages following LPS/ATP treatment (Figure 8C and 8D), conditions that are known to produce high levels of ROS [80].

The lysosome rupture model was tested by the use of cathepsin B inhibitors (Figure 9). Both inhibitors reduced IL-1β secretion and caspase-1 activation in KIM5-infected macrophages. Halle et al. studied activation of the NLRP3 inflammasome in response to phagocytosis of amyloid-beta and showed reduced secretion of IL-1β in cathepsin B knockout macrophages as well as in cathepsin B inhibitor-treated wild type cells [81]. However, off target effects of the inhibitors on caspase-1 activation in KIM5-infected macrophages cannot be ruled out. We could show that the inhibitors reduced IL-1β secretion but not cell death in *S.* Typhimurium infected macrophages undergoing pyroptosis (Figure S2). Therefore, cathepsin B may be specifically required for activation of inflammasomes that are dedicated to processing of pro-IL-1β [19].

In Brodsky et al. [47], *Y. pseudotuberculosis* ectopically expressing YopP was more attenuated than the same strain expressing YopJ^YPTB^ in a mouse oral infection model. The authors suggested that the hypercytotoxic strain eliminated infected macrophages that served as a niche for *Yersinia* survival *in vivo* [47]. Another study showed that a *Y. pestis* strain ectopically expressing YopP was attenuated in a mouse bubonic infection model [49]. In addition, mice infected with the hypercytotoxic attenuated strain were protected against concurrent challenge with fully virulent *Y. pestis* [49]. We hypothesized that the highly cytotoxic YopP could stimulate efficient caspase-1 activation *in vivo* leading to caspase-1-based protection. In an *in vitro* macrophage infection, *Y. pseudotuberculosis* expressing YopP, the same strain used in a previous study [47] triggered high levels of secreted IL-1β and cytotoxicity (Figure 10A and 10B). However, there was no difference in survival for wild type and caspase-1 knockout mice infected with the hyercytotoxic *Y. pseudotuberculosis* strain (Figure 11). Thus, it seems that caspase-1 does not protect mice from oral infection with *Yersinia* strains with enhanced cytotoxicity. Zauberman et al. showed that increased protection of mice against a hypercytotoxic *Y. pestis* strain was seen in subcutaneous challenge, but not in intranasal or intravenous infection, revealing that infection route is important [49]. Our findings do not rule out the possibility that caspase-1 activation is important for protection of mice against subcutaneous infection with a hypercytotoxic *Y. pestis*. In addition, caspase-1-mediated protection of mice against oral challenge with a hypercytotoxic strain may not be measurable using a survival assay, but could significantly impact organ burdens and serum cytokine levels.

In summary, in this paper, we studied the mechanism of caspase-1 and cell death mediated by YopJ^KIM^ and tried to find the relationship between them. Our results indicate that macrophages died by necrosis rather than apoptosis. Caspase-1 activation through the NLRP3/ASC inflammasome may result from K^+^ efflux and lysosome rupture that occur during necrosis. Furthermore, most of the active caspase-1 is located in necrotic cells, and levels of secreted IL-1β could be positively correlated to levels of YopJ/P cytotoxicity. According to the evidence, we hypothesize that necrosis may activate caspase-1 in KIM5-infected macrophages though cathepsin B leaking from lysosome and K^+^ efflux.

## Materials and Methods

### Ethics Statement

All animal use procedures were conducted following the NIH Guide for the Care and Use of Laboratory Animals and performed in accordance with institutional regulations after review and approval by the Institutional Animal Care and Use Committee at Stony Brook University.

### Bacterial strains and growth conditions

The *Y. pestis* strains used in this study, KIM5 and KIM5 expressing YopJ^C172A^, lack the chromosomal pigmentation locus (*pgm*) and are exempt from select agent guidelines [35]. The pACYC184 plasmids encoding *Y. enterocolitica* 8081 YopP or *Y. pseudotuberculosis* IP2666 YopJ (termed as YopJ^YPTB^) were a kind gift of Dr. Igor Brodsky [47]. Condon changes were introduced into the plasmid encoding YopJ^YPTB^ to yield YopJ^KIM^ (F177L) or into the plasmid encoding YopP to yield YopP^C172A^ (C172A) using Quikchange (Invitrogen). *Y. pseudotuberculosis* strain IP2666*ΔyopJ* (termed as IP26) [35] was transformed with pACYC184 or pACYC184 plasmids encoding the different YopP or YopJ isoforms. Plasmid transformation of IP26 was achieved by electroporation, followed by selection on Luria Broth (LB) plates containing chloramphenicol (30 µg/ml) [35]. Cultures of *Y. pestis* and *Y. pseudotuberculosis* for macrophage infection were prepared as described [35].

*S.* Typhimurium SL1344 culture was prepared as described [82]. Briefly, overnight culture was diluted 1∶15 in LB supplemented with 0.3M NaCl and grown at 37°C for 3 hr without shaking.

### BMDM isolation and culture conditions

BMDMs were isolated from bone marrow taken from femurs of 6- to 8-week old C57BL/6J female mice (Jackson Laboratories) as previously described [83]. Frozen stocks of bone marrow cells from C57BL/6 mice deficient for Bax and Bak (Bax^−/−^Bak^−/−^) or heterozyous (Bax^+/−^Bak^+/−^) [84] (obtained from Tullia Lindsten, University of Pennsylvania and Craig Roy, Yale University) were propagated in DMEM GlutaMax supplemented with 20% fetal bovine serum, 30% L-cell-conditioned medium and 1% 0.1 M sodium pyruvate (BMM-high) to obtain BMDM.

### Macrophage infection

Twenty-four hours before infection, BMDMs were seeded in 24-well plates at a density of 1.5×10^5^ cells/well in DMEM GlutaMax supplemented with 10% fetal bovine serum, 15% L-cell-conditioned medium and 1% 0.1 M sodium pyruvate (BMM-low). The next day, macrophages were infected with *Yersinia* at a MOI of 10 as described [35]. For SL1344 infection, cells were infected at an MOI of 10 without a centrifugation step. Gentamicin (15 µg/ml) was added 2 hr post infection, and culture medium was collected following a 2 hr incubation. In some experiments, cells were treated with 10 mM of NAC (Sigma), or 10 µM of DPI (Sigma) for 2 hr before infection. In other experiments the BMDMs were treated with 30 µM of necrostatin-1 (Biomol), 25 µM of CA-074 Me (Biomol), 40 µM of IETD-CHO (Calbiochem) or 25 µM of E64d (Biomol) for 1 hr before infection and during the remainder of the infection period. For the caspase-8 inhibitor positive control experiment, cells were pretreated with 5 µM of MG-132 (Sigma) for 30 min with or without 40 µM of IETD-CHO and then incubated with 1 µg/ml of LPS for 3 hours.

### Microscopic assay to detect surface staining with annexin V and PI uptake

BMDMs were plated on glass coverslips in 24-well plates and infected as described above. At 4, 8 and 12 hr post infection, Annexin V and PI were diluted in Hank's balanced salt solution (HBSS) according to manufacturer's protocol (Roche) and added to the cells. After 15 minutes of staining, the reagents were removed and cells were washed with phosphate buffered saline (PBS). Cells were maintained in PBS and visualized by fluorescence microscopy using a Zeiss Axiovert S100 microscope equipped with a 40× objective. Images were captured using a Spot camera (Diagnostic Instruments, Inc.) and processed by Adobe Photoshop 7.0. Quantification of percent caspase-1 positive BMDMs was performed by scoring macrophages for positive signal in three different randomly selected fields (∼70–130 cells per field) on a coverslip.

### Microscopic assay to detect PI uptake and active caspase-1

BMDMs were plated on glass coverslips in 24-well plates and infected as described above. Nine hours post-infection, macrophages were stained with 6-carboxyfluorescein–YVAD– fluoromethylketone (FAM-YVAD-FMK; Immunochemistry Technologies) as described before [35] and 1 µg/ml PI immediately before observation. Cells were maintained in PBS and visualized by phase and fluorescence microscopy. Images were captured and processed as mentioned above. Quantification of caspase-1 positive or PI positive cell percentages was performed by counting for positive cells in randomly selected fields (∼100–300 cells/field) from three independent experiments.

### Caspase-3/7 luminol assay

Caspase 3/7 activity was measured by Caspase-Glo 3/7 Assay Kit (Promega) according to manufacturer's instruction. BMDMs were seeded in a 96-well white-walled plate at a concentration of 10^4^cells/well in 100 ul medium. Infection was performed as described above. At each time point, a 100 ul of detection buffer was added to a well and the plate was read using a luminescence reader (SpectraMax M2, Molecular Devices).

### Immunoblot analysis

For detection of HMGB1 and PARP by immunoblotting, macrophages were infected as above except that BMDMs were seeded in 6-well plates at a concentration of 10^6^cells/well. Infected cells were maintained in 1 ml of culture medium per well supplemented with 4.5 µg/ml of gentamicin for 24 hours. For detection of HMGB1, harvested culture medium was centrifuged, the supernatant mixed with the same volume of 2×Laemmli buffer, and boiled samples were resolved by SDS-PAGE (15% gels). BMDMs were lysed in 1×Laemmli buffer to obtain a sample of lysate for use as a positive control. Cell lysates for PARP immunoblotting were prepared by removing the media overlaying BMDM monolayers and adding 1×Laemmli buffer into the wells. Boiled lysate samples were resolved SDS-PAGE (8% gels). Uninfected BMDMs were treated with staurosporine (1 µM, Biomol) 16 hours before lysis to provide a control for cleaved PARP. Proteins were transferred from gels to polyvinylidene fluoride (PVDF) membranes and the membranes were probed sequentially with rabbit anti-HMGB1 (Abcam) or anti-PARP (Santa Cruz) primary antibodies, and goat anti-rabbit HRP conjugated secondary antibody (Jackson). Signals on blots were detected with enhanced chemiluminescence reagents (Perkin Elmer Life Sciences, Inc.).

### IL-1β ELISA

IL-1β was measured from supernatants by ELISA [35] according to manufacturer's instructions (R&D).

### LDH Release

Cell death was determined by CytoTox-96 nonradioactive cytotoxicity assay (Promega) from supernatants following manufacturer's instructions. The total LDH release was made by freezing and thaw untreated cells. The percentage of dead cells was calculated as follows: (sample LDH - background LDH)/(total LDH-background LDH)×100%.

### Mouse infection assay

Caspase-1-deficient (Casp1*^−/−^*) mice on the C57BL/6 background [85] were obtained from Richard Flavell and Craig Roy, Yale University. The Casp1*^−/−^* mice upon receipt had been backcrossed to C57BL/6 mice for 7 generations. The Casp1*^−/−^* mice were backcrossed to C57BL/6J mice (Jackson Laboratories) for an additional three generations. The offspring were mated to generate colonies of Casp1*^−/−^* or Casp1*^+/+^* mice that were used for infection at 8–10 weeks of age. *Y. pseudotuberculosis* cultures were grown overnight with shaking in LB at 26°C. Bacteria were harvested by centrifugation and resuspended in PBS. Male and female mice were fasted for 14–16 hr prior to infection. Infection was achieved orogastrically with 1×10^9^ colony forming units of bacteria in 0.2 ml of HBSS using a 20-gauge feeding needle. Mice were monitored three times a day for 21 days. Mice displaying severe signs of disease and deemed unable to survive were euthanized by CO_2_ asphyxiation.

### Statistical analysis

Statistical analysis was performed with Prism 4.0 (Graphpad) software. The tests used are as indicated in the figure legends or main text. *P* values of less than 0.05 were considered significant.

## Supporting Information

Figure S1 Transfer of media from KIM5-infected macrophages to uninfected macrophages does not lead to increased cell death or IL-1β release. BMDMs in 6-well plates with 3 ml of medium per well were infected with *Y. pestis* expressing YopJ^KIM^ or YopJ^C712A^ or left infected (U). Twenty four hours post-infection, supernatants (1 ml) were collected and transferred into wells of a 24 well dish containing uninfected BMDMs or empty wells as background (B) control. Supernatants were collected after an addition 24 hours. IL-1β and LDH were measured by ELISA (panel A) or CytoTox96 assay (panel B), respectively. Results are averaged from three independent experiments and error bars represent standard deviations.
(TIF)

Figure S2 Cathepsin B inhibitors reduced IL-1β release, but not cell death in macrophages infected with *S.* Typhimurium SL1344. BMDMs were left untreated or pretreated with 25 µM of E64d or CA-074 Me (CA) for 1 hr. Untreated BMDMs were left uninfected (U) or infected with SL1344 at an MOI of 10 for 4 hours. Treated BMDMs were infected with SL1344 under the same conditions in the presence of the inhibitors. Medium was collected for IL-1β ELISA (A) and LDH release assays (B). Results shown are the average of two independent experiments. Error bars represent standard deviations.
(TIF)
