# Supplementary figures and images for: YopJ-Induced Caspase-1 Activation in Yersinia-Infected Macrophages: Independent of Apoptosis, Linked to Necrosis, Dispensable for Innate Host Defense
Source: PLoS One. 2012 Apr 26;7(4):e36019. doi: 10.1371/journal.pone.0036019 (PMC3338577; doi:10.1371/journal.pone.0036019)

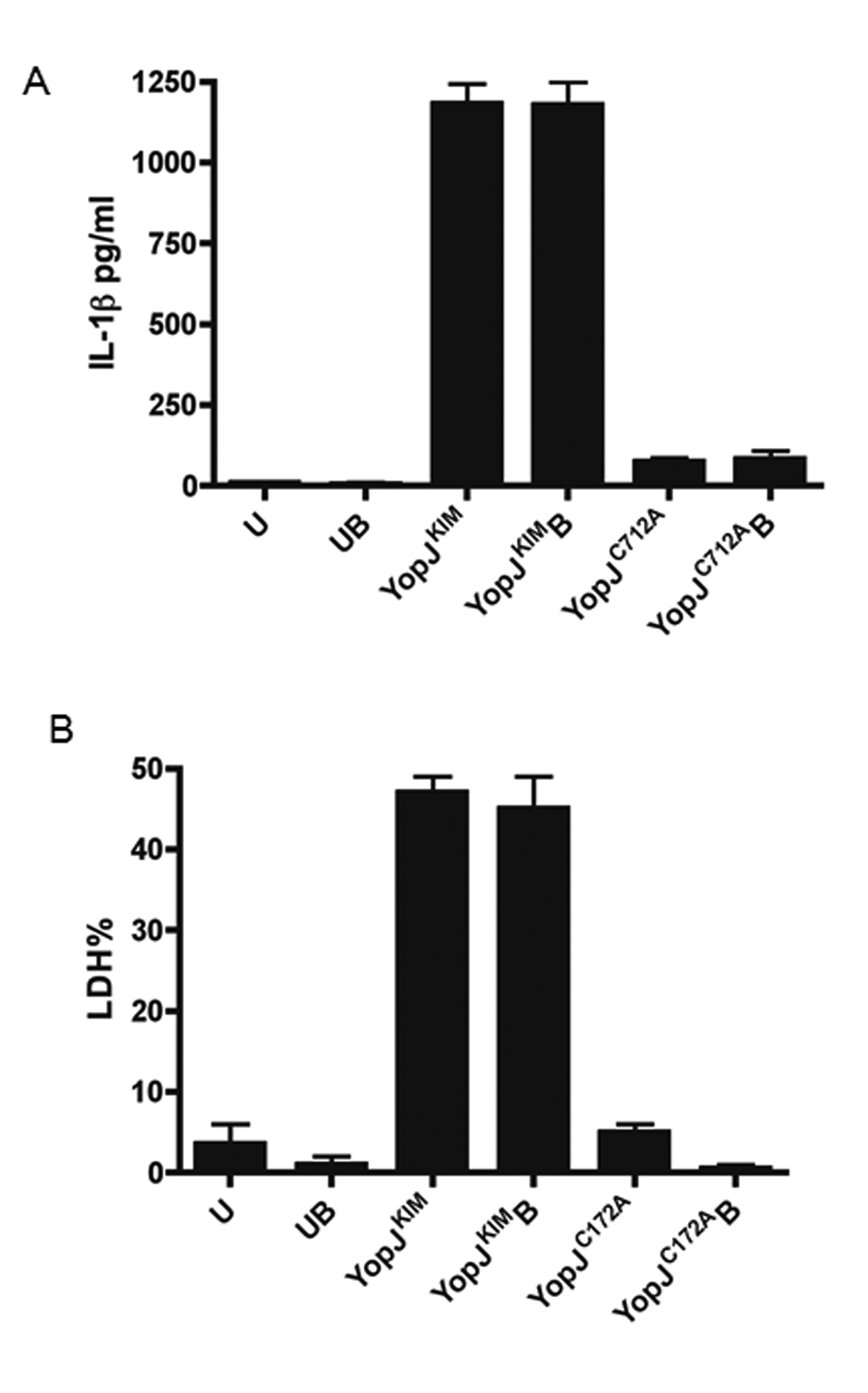

Supplement: Figure S1 — Transfer of media from KIM5-infected macrophages to uninfected macrophages does not lead to increased cell death or IL-1β release. BMDMs in 6-well plates with 3 ml of medium per well were infected with Y. pestis expressing YopJKIM or YopJC712A or left infected (U). Twenty four hours post-infection, supernatants (1 ml) were collected and transferred into wells of a 24 well dish containing uninfected BMDMs or empty wells as background (B) control. Supernatants were collected after an addition 24 hours. IL-1β and LDH were measured by ELISA (panel A) or CytoTox96 assay (panel B), respectively. Results are averaged from three independent experiments and error bars represent standard deviations. (TIF) [file pone.0036019.s001.tif]

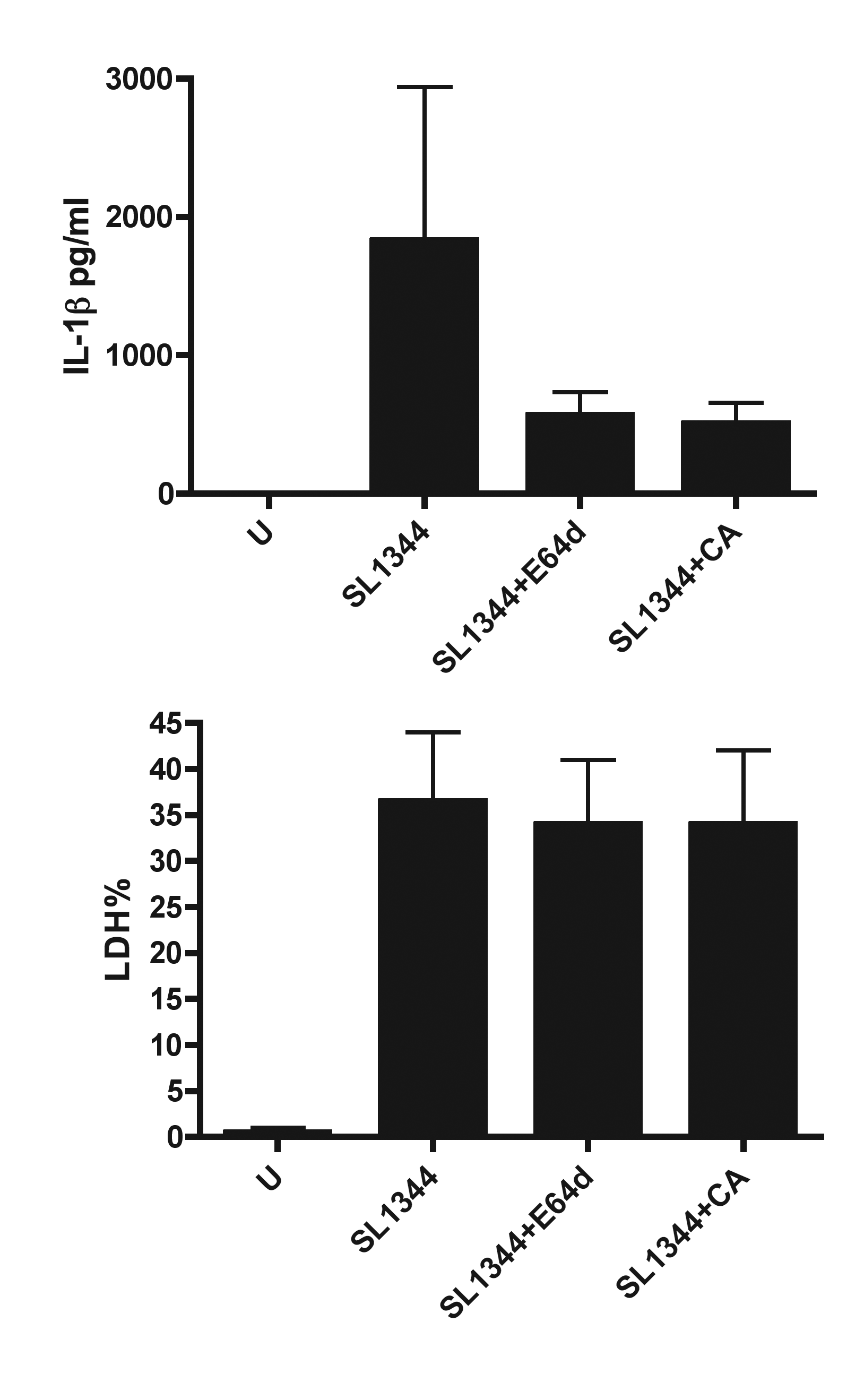

Supplement: Figure S2 — Cathepsin B inhibitors reduced IL-1β release, but not cell death in macrophages infected with S. Typhimurium SL1344. BMDMs were left untreated or pretreated with 25 µM of E64d or CA-074 Me (CA) for 1 hr. Untreated BMDMs were left uninfected (U) or infected with SL1344 at an MOI of 10 for 4 hours. Treated BMDMs were infected with SL1344 under the same conditions in the presence of the inhibitors. Medium was collected for IL-1β ELISA (A) and LDH release assays (B). Results shown are the average of two independent experiments. Error bars represent standard deviations. (TIF) [file pone.0036019.s002.tif]
